# Neuronal Response to Reward and Luminance in Macaque LIP During Saccadic Choice

**DOI:** 10.1007/s12264-022-00948-0

**Published:** 2022-09-17

**Authors:** Ziqi Wu, Aihua Chen, Xinying Cai

**Affiliations:** 1 Key Laboratory of Brain Functional Genomics (Ministry of Education), East China Normal University (ECNU), Shanghai, 200062 China; 2 New York University (NYU) Shanghai, Shanghai, 200122 China; 3 NYU-ECNU Institute of Brain, Cognitive Science at NYU Shanghai, Shanghai, 200062 China; 4 Key Laboratory of Brain Functional Genomics (Ministry of Education), School of Psychology and Cognitive Science, ECNU, Shanghai, 200062 China

**Keywords:** Reward, Luminance, Attention, Priority map, Saccadic choice, Lateral intraparietal cortex

## Abstract

Recent work in decision neuroscience suggests that visual saliency can interact with reward-based choice, and the lateral intraparietal cortex (LIP) is implicated in this process. In this study, we recorded from LIP neurons while monkeys performed a two alternative choice task in which the reward and luminance associated with each offer were varied independently. We discovered that the animal’s choice was dictated by the reward amount while the luminance had a marginal effect. In the LIP, neuronal activity corresponded well with the animal’s choice pattern, in that a majority of reward-modulated neurons encoded the reward amount in the neuron’s preferred hemifield with a positive slope. In contrast, compared to their responses to low luminance, an approximately equal proportion of luminance-sensitive neurons responded to high luminance with increased or decreased activity, leading to a much weaker population-level response. Meanwhile, in the non-preferred hemifield, the strength of encoding for reward amount and luminance was positively correlated, suggesting the integration of these two factors in the LIP. Moreover, neurons encoding reward and luminance were homogeneously distributed along the anterior-posterior axis of the LIP. Overall, our study provides further evidence supporting the neural instantiation of a priority map in the LIP in reward-based decisions.

**Supplementary Information:**

The online version contains supplementary material available at 10.1007/s12264-022-00948-0.

## Introduction

Recent work in decision neuroscience suggests that visual saliency may interact with the reward-based decision process [1, 2]. Multiple lines of evidence suggest that the lateral intraparietal cortex (LIP) is a candidate area where visual saliency and reward interact during saccadic choice [3–5]. Early work leading to the proposal of the LIP acting as a priority map showed in separate studies that visually-responsive neurons in the LIP change their firing rates in response to both bottom-up saliency and top-down goals [6–9]. A parallel line of studies has shown that LIP activity is modulated by reward or subjective value based on which saccadic choices are made [10–17]. Moreover, LIP neurons can automatically encode visual salience [3, 5, 18], which may have significant implications for the effect of visual saliency on economic choice [1, 2]. However, to the best of our knowledge, no studies have examined whether or how single LIP neurons integrate visual saliency and reward in a choice setting.

Adding to the complexity of this question is the increasing evidence that the LIP is not a functionally homogeneous area. Non-human primate studies suggest that dorsal-ventral subdivisions of the LIP may be preferentially involved in different aspects of visual-oculomotor processes [19, 20]. Increasing evidence also points to potential subdivisions along the caudal-rostral extent of the LIP [21–23]. These results highlight the urgency for broader sampling when examining the contribution of the LIP to saccadic choice using single-unit recordings.

To address these issues, we performed an unbiased sampling of LIP neurons covering its anterior-posterior (AP) extent while monkeys performed a two alternative choice task in which the reward and luminance associated with each offer were varied independently. We report the following primary results. First, the animal’s choice was dictated by the reward amount while the luminance had a marginal effect. Second, the LIP neuronal response to reward and luminance corresponded well with the animal’s choice pattern. Third, neurons encoding reward and luminance were evenly distributed along the AP axis of the LIP.

## Materials and Methods

### Experimental Preparation

Two male rhesus monkeys (C, 7.5 kg; F, 8.5 kg; Suzhou Xishan Zhongke Laboratory Animal Co., Ltd., Suzhou, China) were used in the experiments. Each animal was chronically implanted with a circularly molded, lightweight plastic ring for head restraint and a scleral coil for monitoring eye movements within a magnetic field (Crist Instrument Co., MD, USA). All procedures were approved by the Animal Care and Use Committee of East China Normal University.

### Neuronal Recordings

Single-unit activity was recorded from the ventral portion of the lateral bank of the intraparietal sulcus. We used both single-channel tungsten microelectrodes (FHC, Bowdoin, USA) and linear microelectrode arrays (LMA, Microprobes, Gaithersburg, USA). A hydraulic microdrive (FHC) was used to advance the microelectrode into the cortex through a transdural guide tube. The recording grid was parallel to the horizontal plane such that the electrodes were advanced perpendicularly. Neural signals were collected with a multi-channel acquisition system (AlphaLab SnR, Alpha Omega, Nof HaGalil, Israel). All neurons were recorded from the right hemisphere (128 and 180 neurons from monkeys C and F, respectively). Recording locations were localized using a combination of magnetic resonance imaging scans, stereotaxic coordinates, white/gray matter transitions, and physiological response properties. Since we recorded from a linear array in the majority of sessions and often recorded multiple neurons simultaneously, we did not position the visual cues precisely in the receptive field of any particular neuron. Instead, two offers were placed along the horizontal meridian, similar to the placement of two potential goals in Leathers and Olson [24]. Analysis of neural activity in the memory-guided saccade task also confirmed that the direction vectors of LIP neurons recorded here were centered around the horizontal meridian (see Results, Fig. 4).

### Memory-Guided Saccade Task

The animal began each trial by fixating a white fixation point (0.25° × 0.25°) presented at the center of a computer screen. After a 0.5 s fore-period, a circular peripheral visual cue (1.5° × 1.5°) at two possible luminance levels (RGB code [1, 1, 1] or [0.2, 0.2, 0.2]), appeared for 0.2 s at one of eight possible locations on a circle (7° radius) centered on the fixation point. The disappearance of the visual cue was followed by a randomly-selected delay period (0.8–1.1 s, uniform distribution) after which the central fixation point extinguished, and the animal was required to saccade to the peripheral location where the visual cue was previously shown, within 0.4 s. When the animal’s eye trace reached the vicinity of the previously-shown target location, a white target (0.25° × 0.25°) appeared at the same location to serve as a peripheral fixation target. A drop of water (0.125 mL) was delivered after the animal fixated on the peripheral target for 0.3 s. We analyzed the neuronal activity in three non-overlapped time windows—the cue period (200 ms after visual cue presentation), the memory period (middle 400 ms of the 800 ms delay period shared across all trials), and the saccade period (250 ms leading to saccade initiation).

### Choice Task

**Fig. 1:**
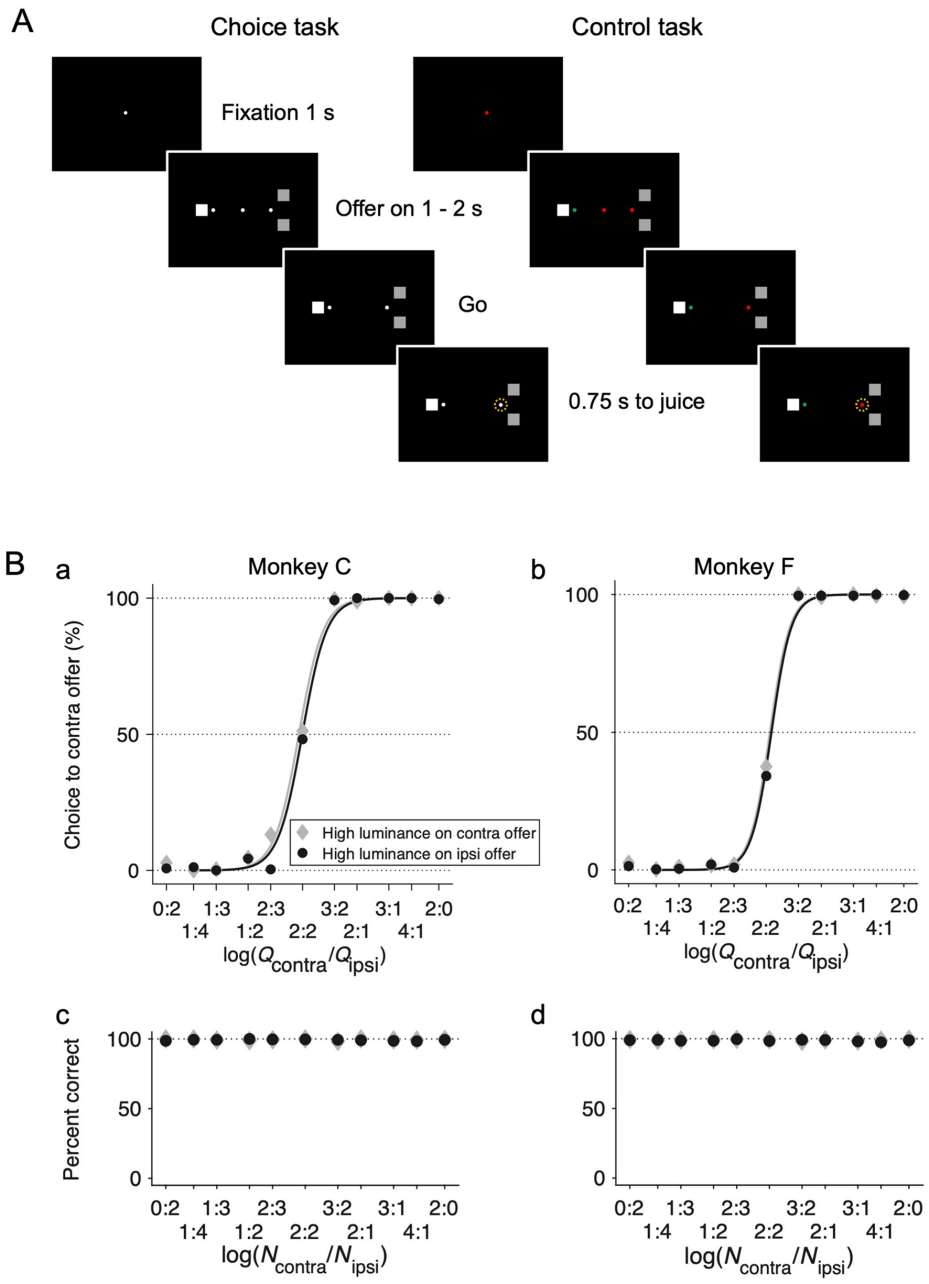
Behavioral tasks. **A** Spatiotemporal sequences of the reward-based choice task and the control task. **B** Choice patterns. **Ba**, **Bb** Summary choice pattern in the choice task for monkey C (38 sessions, 5,626 trials) and monkey F (70 sessions, 10,125 trials). The percentage of contralateral choices is plotted against log(*Q*_contra_/*Q*_ipsi_), where *Q*_contra_ and *Q*_ipsi_ are quantities (0.125 mL per square symbol) of the contra and ipsilateral offer, respectively (note: quantity ratios 0:2 and 2:0 are plotted separately thus not on log scale). Trials were separated into two groups depending on the luminance level for the two offers (filled gray diamonds: high luminance on the contralateral offer, filled black circles: high luminance on the ipsilateral offer). The regression lines were obtained from Eq.1. **Bc**, **Bd** Summary choice accuracy in the control task for monkeys C (38 sessions, 6,325 trials) and F (70 sessions, 10,438 trials). *N*_contra_ and *N*_ipsi_ represent the number of stimulus symbols in the contralateral and ipsilateral hemifield (note: number ratios 0:2 and 2:0 are plotted separately thus not on log scale).

The animal performed a reward-guided choice task (referred to as the choice task) and a control task in alternating blocks. In the choice task (Fig. 1A left column), the animal began each trial by fixating a white fixation point (0.25° × 0.25°) presented at the center of a computer screen. After a 1 s fore-period, two offers represented by two sets of a variable number of squares (1.5° × 1.5°) with high (RGB color code = [1, 1, 1], 7.7 cd/m^2^ per square symbol) or low luminance (RGB color code = [0.2, 0.2, 0.2], 0.37 cd/m^2^ per square symbol) and their associated saccade targets (0.25° × 0.25°, white) appeared on opposite sides of the fixation point (8° along the horizontal meridian). The reward quantity (0.125 mL) of the contralateral and ipsilateral offers were configured as [0:2, 1:4, 1:3, 1:2, 2:3, 2:2, 3:2, 2:1, 3:1, 4:1, 2:0] (reward matrix #1). In a small subset of sessions (11 out of 81) for monkey F, the quantity combinations were [0:1, 1:4, 1:3, 1:2, 1:1, 2:1, 3:1, 4:1, 1:0] (reward matrix #2). The luminance level and the position of the two offers were counter-balanced for each reward quantity combination within a block. The animal was required to shift its gaze toward one of the targets within 0.4 s, when the central fixation point and the two sets of squares were extinguished after a 1–2 s random offer-on period. The animal had to maintain fixation on the target for an additional 0.75 s before reward delivery. The amount of reward for each offer was proportional to the number of squares (0.125 mL/square) regardless of the luminance level of the offers.

### Control Task

To verify that neuronal responses in the choice task were largely driven by reward amount but not the number of visual symbols, we also designed a control task (Fig. 1A right column), which was identical to the choice task, except for the following two changes. First, the central fixation target was either green or red, which instructed the animal to choose the peripheral target of the same color. Second, after choosing the correct target, the animal was always rewarded with a fixed amount of water (0.25 mL), which was equivalent to the reward quantity associated with two squares in the choice task. The animal performed the choice and control tasks in alternating blocks of trials.

### Analysis of Choice Pattern

All analyses were conducted in MatLab (MathWorks, USA). We constructed the logistic model described in equation 1 (Eq.1), which included the reward quantity ratio and luminance level of the two offers,

1 $$ {\text{choice}} \, C_{{{\text{contra}}}} = { 1}/\left( {{1 } + {\text{ e}}^{ - X} } \right) $$

$$ X = {\text{ a}}_{0} + {\text{ a}}_{{1}} {\text{log}}\left( {Q_{{{\text{contra}}}} /Q_{{{\text{ipsi}}}} } \right) \, + {\text{ a}}_{{2}} (\delta_{{{\text{contra}},{\text{ high luminance}}}} - \delta_{{{\text{ipsi}},{\text{ high luminance}}}} ) $$

where choice *C*_contra_ = 1 if the animal chose the contralateral offer and 0 otherwise; *Q*_i_ was the quantity of offer *i* (with *i* = contra or ipsi); *δ*_*i*, high luminance_ = 1 if offer *i* has high luminance and 0 otherwise. The logistic regression was performed on aggregated trials across all sessions. Parameters a_0_ – a_2_ are logistic regression coefficients. The relative value of the two offers was measured by *ρ* = exp(−a_0_/a_1_) and the effect of luminance was quantified with the normalized coefficient *ξ* = a_2_/a_1_.

### Regression Analysis for Neuronal Activity

For a total of 308 neurons, stable recording was achieved for more than one experimental block (96 trials for reward matrix #1; 80 trials for reward matrix #2) during the choice task and thus were included in the database for analysis. Among these neurons, 301 were tested in at least two blocks of choice trials. The regression analysis was applied to trials in which the animal chose the offer in the contralateral hemifield, and the average number of trials in the choice task tested for each neuron was 90 ± 25 (mean ± SD). The regression model included the reward amount on the contralateral (Rew_contra_) and ipsilateral hemifield (Rew_ipsi_), the luminance level of the offer in the contralateral hemifield (Lum, 0 and 1 for low and high luminance levels), and the animal’s choice (Cho, 0 and 1 for choice to ipsilateral and contralateral side):

$$ S =\upbeta _{0} +\upbeta _{{1}} \times {\text{ Rew}}_{{{\text{contra}}}} +\upbeta _{{2}} \times {\text{ Rew}}_{{{\text{ipsi}}}} +\upbeta _{{3}} \times {\text{ Lum}} +\upbeta _{{4}} \times {\text{ Cho }}\left( {\text{model 1}} \right) $$

where *S* denotes the spike rate during the post-offer period (500 ms after offer on). The dependent and independent variables were *Z*-scored before applying the regression analysis such that β_1_–β_4_ represented the standardized regression coefficients (SRCs). The statistical significance of each regression coefficient was determined with a *t*-test (*P* <0.05). Since there were only two levels of luminance for each offer and the luminance levels for the two offers were always anti-correlated, the term Lum essentially captured the effect of luminance contrast between the contralateral and ipsilateral hemifields. Note that in the regression analysis for Fig. 7B, the high/low luminance level in the non-preferred hemifield was coded as 1/0.

We applied the same model to the neuronal activity in the control task:

$$ S =\upbeta _{0} +\upbeta _{{1}} \times {\text{ Num}}_{{{\text{contra}}}} +\upbeta _{{2}} \times {\text{ Num}}_{{{\text{ipsi}}}} +\upbeta _{{3}} \times {\text{ Lum}} +\upbeta _{{4}} \times {\text{ Cho }}\left( {\text{model 2}} \right) $$

In this model, we replaced Rew_contra_/Rew_ipsi_ with Num_contra_/Num_ipsi_ and other variables remained the same.

To identify neurons modulated by saccades, we applied model 2 to the spike rate during the 500-ms window leading to the initiation of a saccade in the control task. Neurons with significant β_4_ were categorized as the saccade-modulated group while the rest belonged to the non-saccade-modulated group. In the modulated group, for neurons with β_4_ >0, we considered the contralateral hemifield as their preferred hemifield while for those with β_4_ <0, the ipsilateral hemifield was considered the preferred hemifield.

We used the coefficient of partial determination (CPD) to quantify how strongly neural activity was influenced by each of the regressors. We performed the analysis using a 200-ms window shifted in 25-ms steps. The raw CPD for *X*_*i*_ was defined as follows:

$$ {\text{CPD}}_{{{\text{raw}}}} \left( {X_{i} } \right) = \left\{ {{\text{SSE}}\left( {X_{ - i} } \right){-}{\text{SSE}}\left( X \right)} \right\}/{\text{SSE}}\left( {X_{ - i} } \right), $$

in which SSE(*X*) refers to the sum of squared errors in a regression model that includes a full set of regressors *X* while SSE(*X*_−*i*_) (note the minus sign in front of *i*) refers to the sum of squared errors in the same regression model including the full set of regressors except for *X*_*i*_. To compare CPDs across choice and control tasks, for each neuron and each task, we computed the CPD based on the same set of trials with the trial label for the dependent variable randomly shuffled and we repeated the shuffling procedure 1000 times. The mean CPD from the shuffling procedure was considered the CPD for the baseline condition (CPD_baseline_). The CPD with baseline removed for each independent variable was defined as CPD(*X*_*i*_) = CPD_raw_(*X*_*i*_) – CPD_baseline_(*X*_*i*_).

## Results

### Choice Task Design and Choice Patterns

Fig. 1A illustrates the experimental design. In the choice task (Fig. 1A, Choice Task), we varied the reward amount and luminance level of two offers independently on a trial-by-trial basis with the number of symbols representing reward amount and the stimulus luminance level a reward-independent but perceptually salient visual feature. To verify that the neuronal responses were largely driven by reward amount but not the number of visual symbols, we also designed a control task in which visual presentations of the offers remained the same but the number of symbols no longer covaried with the reward amount. To make a correct choice, the animal must saccade to the target with a color matching that of the central fixation point (Fig. 1A, Control Task). Fig. 1B shows the summary choice pattern based on the aggregated trials across all sessions for monkeys C and F. Trials were divided into two groups depending on whether the high luminance offer was in the contralateral or ipsilateral hemifield. For a quantitative analysis of choice patterns, we constructed a logistic model that included the reward quantity ratio and luminance level associated with the offers (see Materials and Methods, Eq. 1). In the majority of sessions (all 38 sessions for monkey C and 70 out of 81 sessions for monkey F), the reward quantity (0.125 mL) of the two offers followed the combinations of *Q*_contra_:*Q*_ipsi_ = [0:2, 1:4, 1:3, 1:2, 2:3, 2:2, 3:2, 2:1, 3:1, 4:1, 2:0]. We performed a logistic analysis of each animal based on the aggregated trials (see Materials and Methods).

As demonstrated in Fig. 1Ba and Bb, the reward ratio (*ρ*) significantly influenced the choice of both animals (monkey C: *ρ* = 0.9891, *P* <10^−10^; monkey F: *ρ* = 1.0600, *P* <10^−10^). On the other hand, in both monkeys, luminance (*ξ*) had a small effect on the choice pattern (monkey C, *ξ* = 0.0245, *P* = 0.13; monkey F, *ξ* = 0.0218, *P* = 0.043). Translating the value of *ξ* to the choice pattern, this means that having high luminance on the ipsilateral offer was equivalent to multiplying the quantity of the offer by a factor of exp (*ξ*), which was 1.0248 [exp(0.0245)] for monkey C and 1.0220 [exp(0.0218)] for monkey F, indicating a marginal effect.

In the control task, the animal was instructed by the color of the central fixation target to make a saccade to the peripheral target of the same color. Among all trials, monkeys C and F chose the correct target 97.42% and 98.69% of the time, respectively (Fig. 1Bc, Bd).

In a subset of sessions (11 out of 81) with monkey F, the reward quantity combinations followed reward matrix #2 (*Q*_contra_:*Q*_ipsi_ = [0:1, 1:4, 1:3, 1:2, 1:1, 2:1, 3:1, 4:1, 1:0]) and the animal only split its choice for the equal reward condition (Fig. S1A). In these sessions, the logistic fit for aggregated trials did not reach convergence because the slope of the logistic function could not be accurately estimated. We thus applied an alternative analysis. We sorted trials according to the reward ratio between the high and low luminance offer and then applied a binomial test to determine whether the choice probability significantly deviated from 0.5. We found that in 2 of these 11 sessions, the animal tended to prefer the high luminance offer (*P* <0.05, binomial test) while there was no significant difference in the choice probability for the high or low luminance offer in the remaining 9 sessions (*P* >0.05, binomial test).

In summary, in the choice task, the reward amount dictated the animals’ choice while luminance generated a marginal effect; in the control task, the animal chose with high accuracy according to the color-matching rule.

### Characterizing Neuronal Responses in the Memory-Guided Saccade Task

**Fig. 2:**
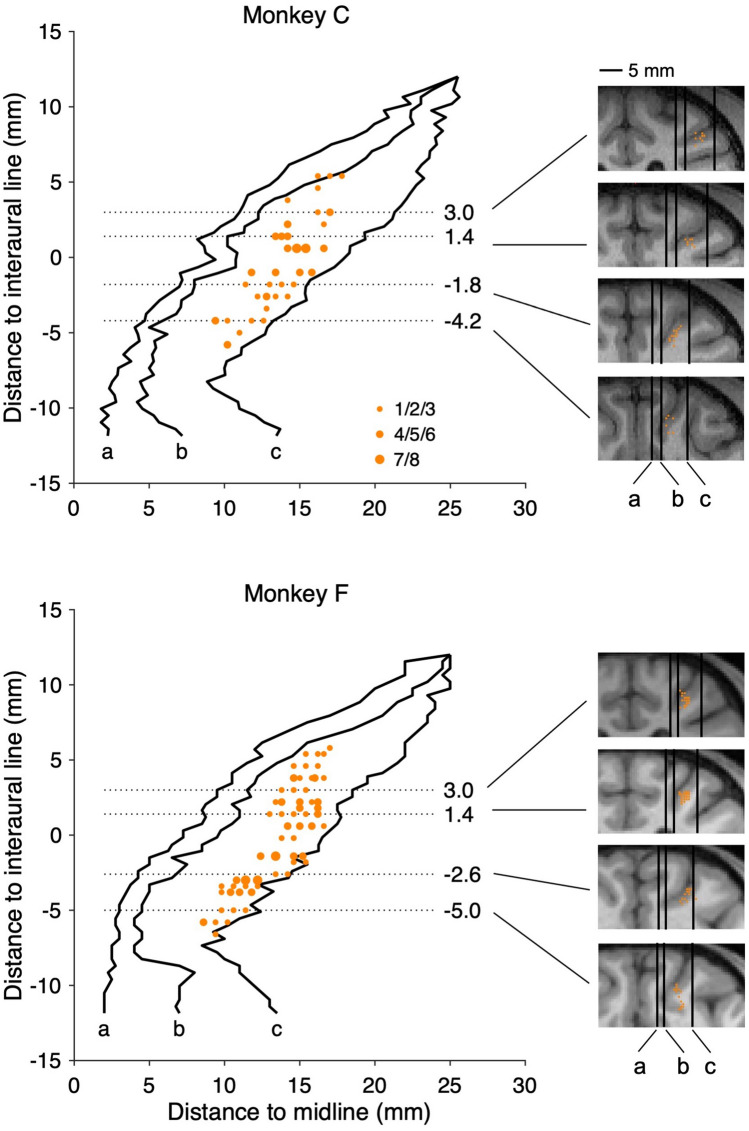
Recording locations. Locations of neurons recorded in the LIP of two animals projected onto the horizontal plane. The size of each symbol indicates the number of neurons recorded from that location. The outlines obtained from MR images correspond to the medial limit of the fundus of the intraparietal sulcus (IPS) (a), the medial limit of the IPS (b), and the lateral limit of the LIP (c). For each animal, four representative MR coronal images are shown and the recording locations are projected to the nearest image according to the distance along the anterior-posterior axis.

**Fig. 3:**
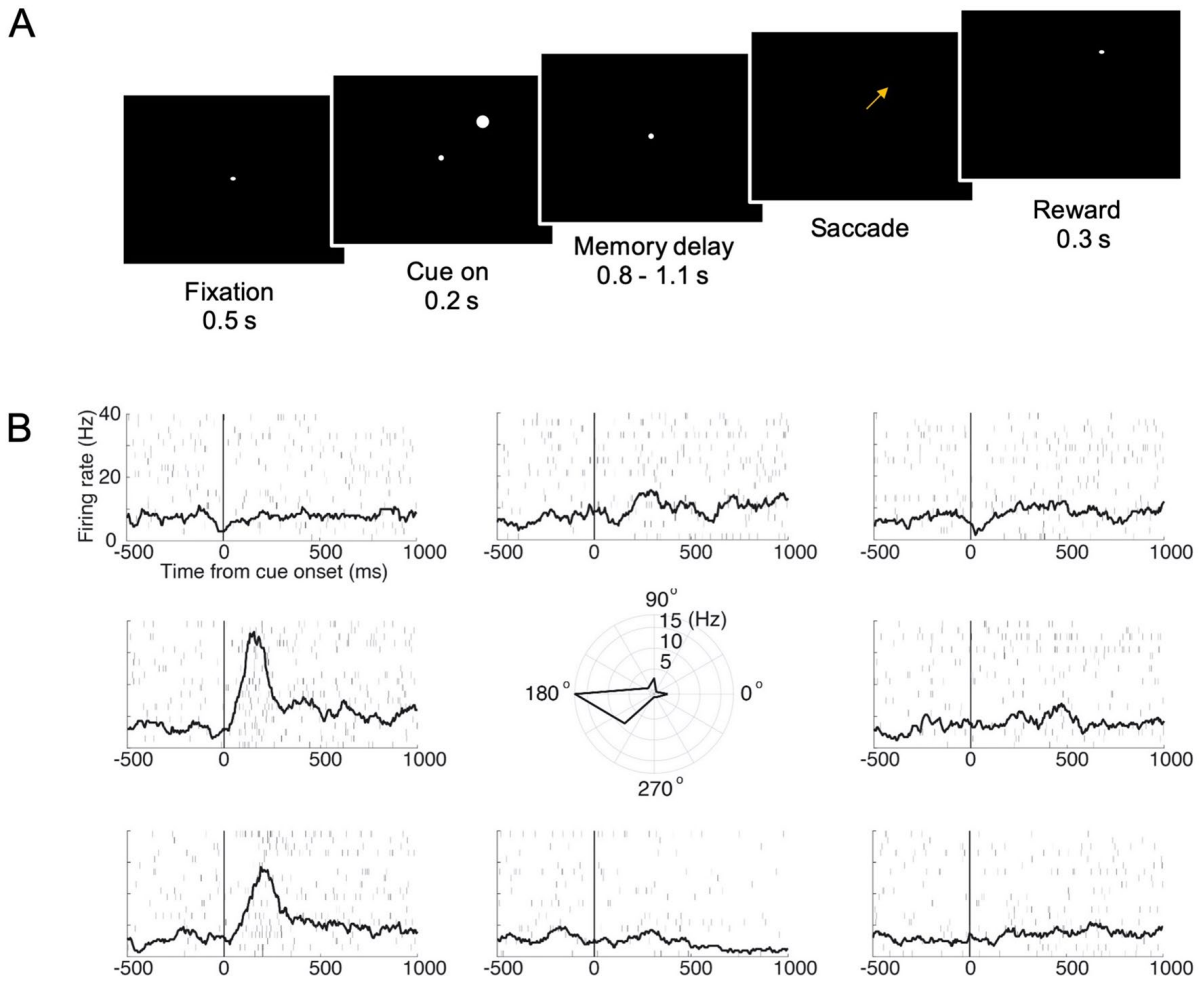
Example neuronal responses in the memory-guided saccade task. **A** Spatiotemporal sequences of the memory-guided saccade task. **B** Raster and mean spike density functions of neuronal responses from one representative neuron plotted separately according to saccade target locations. The central polar plot shows the directional tuning of neuronal responses during the cue period.

In total, 308 recorded neurons (Fig. 2) were included in the analysis for the choice task, out of which we also examined the response properties of 179 cells with a memory-guided saccade (MGS) task (Fig. 3A). We remind that in this study, we aimed to perform an unbiased characterization of the response properties of LIP neurons. Thus, in both the MGS and choice task, we recorded every neuron the electrode encountered and analyzed the activity of neurons that had a sufficient number of trials.

**Fig. 4:**
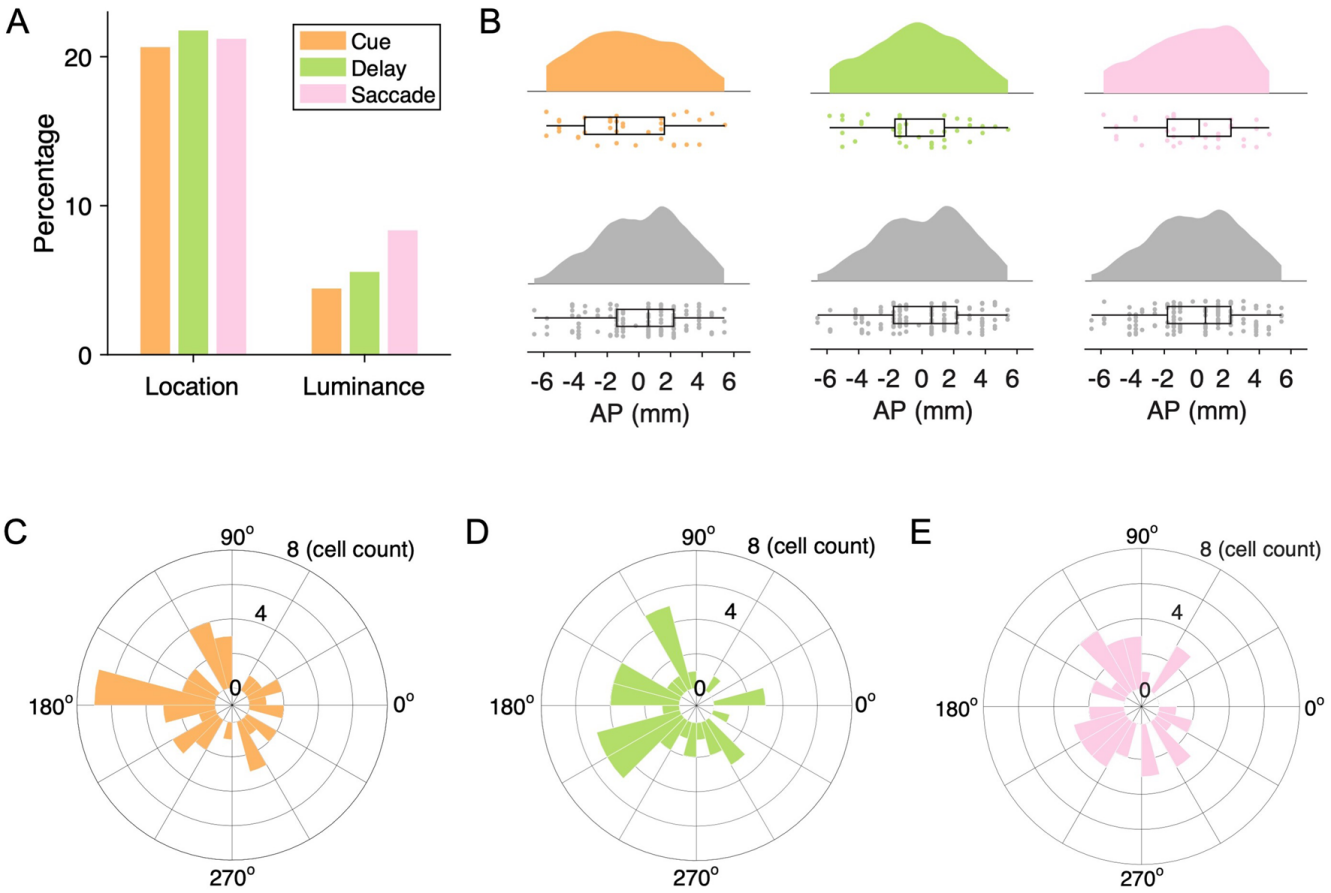
Neuronal coding in the memory-guided saccade task. **A** Percentages of neurons significantly tuned to the location/luminance of the stimulus during the cue (20.7%/4.5%), delay (21.8%/5.6%), and saccade (21.2%/8.4%) periods. **B** Distribution of AP coordinates of neurons tuned (top) and not tuned (bottom) to the stimulus location during the cue (tuned, *n* = 37; not tuned, *n* = 142), delay (tuned, *n* = 39; not tuned, *n* = 140), and saccade periods (tuned, *n* = 38; not tuned, *n* = 141). **C–E** Distributions of direction vectors of spatially-tuned neurons during the cue (**C**), memory (**D**), and saccade (**E**) periods.

Fig. 3B shows the raster and the mean spike density function from one representative neuron in the MGS task, which showed a strong spatial preference for the contralateral hemifield. We tested the spatial and luminance selectivity of all neurons with two-way ANOVA. We found that during the cue period, 20.7% (37/179, above chance level, *P* <10^−12^, binomial test) and 4.5% (8/179, not above chance level, *P* = 0.68, binomial test) of the neurons were spatial and luminance selective; during the memory period, the percentages of spatial and luminance selective neurons were 21.8% (39/179, above chance level, *P* <10^−14^, binomial test) and 5.6% (10/179, not above chance level, *P* = 0.41, binomial test), while in the saccade period, the percentages of spatial- and luminance-selective neurons were 21.2% (38/179, above chance level, *P* <10^−13^, binomial test) and 8.4% (15/179, above chance level, *P* <0.05, binomial test), respectively (Fig. 4A). We compared the distribution of anterior-posterior (AP) coordinates of neurons demonstrating spatial tuning with those that were not spatially tuned and did not find any difference between the distribution in the cue [*P* = 0.27, Kolmogorov-Smirnov (KS) test], memory (*P* = 0.89, KS test), or saccade period (*P* = 0.96, KS test) (Fig. 4B). The direction vectors of neurons that demonstrated spatial selectivity were skewed towards the contralateral hemifield (Fig. 4C, cue period, mean = 171.5°, *P* = 0.067, Rayleigh test; Fig. 4D, memory period, mean = 236.8°, *P* <0.01, Rayleigh test; Fig. 4E, saccade period, mean = 206.2°, *P* = 0.13, Rayleigh test).

### Neuronal Activity Related to Reward and Luminance

**Fig. 5:**
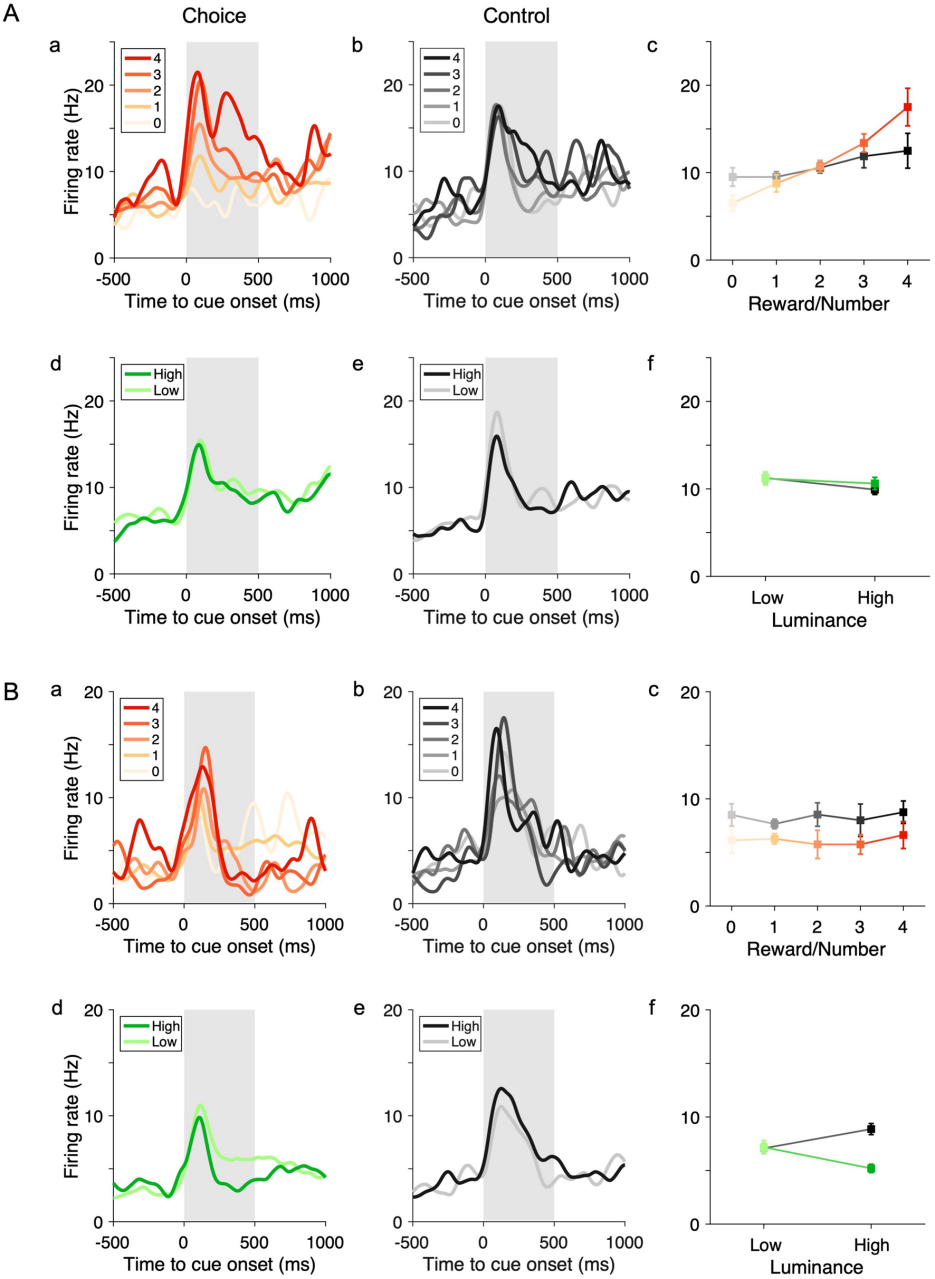
Example neurons encoding task-related variables. **A** A neuron encoding the reward amount in the choice task. **Aa, Ab** Spike density functions in the choice (**Aa**) and control (**Ab**) tasks. **Ac** Firing rates during the post-offer period averaged according to the reward amount in the choice task (color scale) and the number of symbols (grayscale) in the control task. The gray rectangle covers the post-offer period. **Ad–f** As in **Aa–c** but the neuronal activity was grouped according to the luminance level in the contralateral hemifield. **B** As in **A** but for another neuron encoding luminance level. Error bars, SEM.

In the choice task, many neurons were modulated by task variables, particularly the amount of reward and luminance level associated with the stimulus. The effect was tested with regression analysis (models 1 and 2). Fig. 5 shows two representative neurons. The activity of neuron 1 during the post-offer window (0–500 ms after an offer on) increased with the increase of the contralateral reward amount in the choice task (Fig. 5Aa). The increase of activity with the increase of stimulus number in the control task was much less significant (Fig. 5Ab). This result was confirmed with a regression analysis revealing a significant encoding of reward amount in the choice task (Fig. 5Ac, *P* <10^−4^) but not the stimulus number in the control task (Fig. 5Ac, *P* = 0.057). Meanwhile, it was not modulated by the stimulus luminance level in either the choice or the control task (Fig. 5Ad–f, choice task: *P* = 0.51; control task: *P* = 0.10). The activity of neuron 2 during the post-offer window was not significantly modulated by reward amount in the choice task or the stimulus number in the control task (Fig. 5Ba–c, choice task: *P* = 0.56; control task: *P* = 0.62). On the other hand, it was significantly modulated by luminance level in the choice task with a negative slope but in the control task with a positive slope (Fig. 5Bd–f; choice task: *P* < 0.001; control task: *P* < 0.05).

**Fig. 6:**
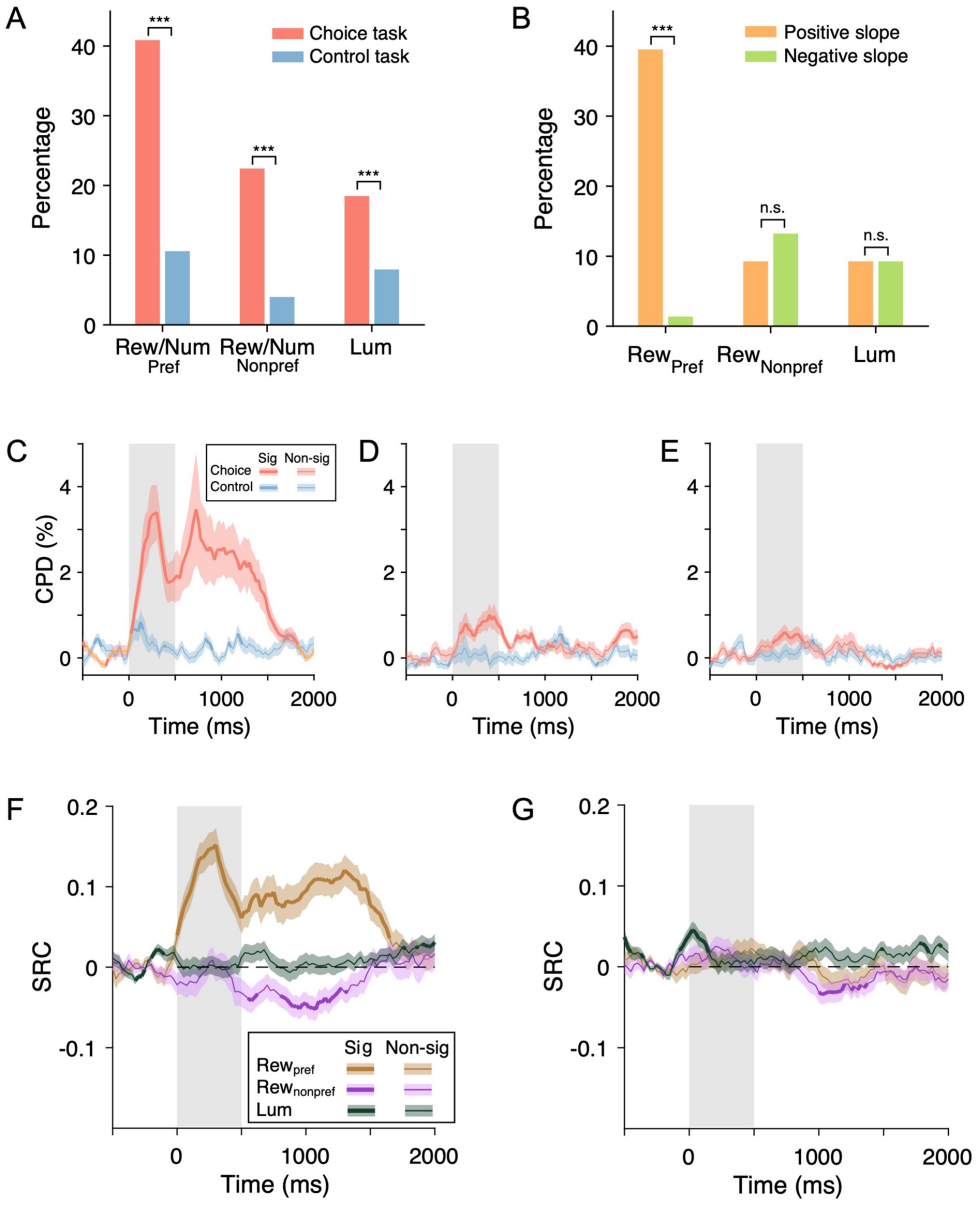
Encoding of task-related variables in the choice and control task among neurons modulated by saccades in the control task. **A** Percentages of cells encoding reward (Rew, choice task) or stimulus number (Num, control task), and luminance level (Lum) (***P <0.001, *Z*-test). Pref, preferred hemifield; Nonpref, nonpreferred hemifield. **B** Percentages of neurons encoding reward and luminance with a positive and negative slope in the choice task (***P <0.001, *Z*-test). **C** Time course of neuronal responses related to reward and luminance. Population average of coefficient of partial determination (CPD) for reward amount/stimulus number in the preferred hemifield in the choice/control task. Each data point was computed based on the firing rate in a sliding time window (width = 200 ms, step = 25 ms). The thick line indicates that the mean CPD is significantly (Sig) above baseline (*P* <0.05, *t*-test). The gray rectangle covers the post-offer window. Shaded regions indicate mean ± SEM. **D** As in **C** but for reward amount/stimulus number in the non-preferred hemifield. **E** As in **C** but for luminance. **F** Time course of the standardized regression coefficients (SRCs) for reward and luminance in the choice task (*n =* 308). Each data point was computed based on the firing rate in a sliding time window (width = 200 ms, step = 25 ms). The thick line indicates that the mean SRC significantly deviates from 0 (*P* <0.05, *t*-test). Shaded regions indicate mean ± SEM. The gray rectangle covers the post-offer window. **G** As in **F** but for stimulus number and luminance in the control task (*n =* 308).

We next computed the percentage of neurons significantly encoding reward and luminance. We first divided the neurons into two groups depending on whether their activity was modulated according to the animal’s choice in the saccade-related window (defined as a 500-ms interval leading to the initiation of a saccade) in the control task. The saccade-modulated group made up 24.7% (76/308) of the recorded population. For this subgroup of neurons, we defined the direction that induced higher activity during the saccade period as the neuron’s preferred side. Overall, 40.8% (31/76), 22.4% (17/76), and 18.4% (14/76) of neurons encoded the reward amount in the preferred hemifield, non-preferred hemifield, and luminance level, respectively, in the choice task (Fig. 6A, all above chance level, *P* <10^−4^, binomial test). In the control task, the percentages of neurons encoding stimulus number in the preferred hemifield, non-preferred hemifield, and the luminance level were 10.5% (8/76, above chance level, *P* <0.05, binomial test), 3.9% (3/76, not above chance level, *P* = 0.74, binomial test), and 7.9% (6/76, *P* = 0.18, binomial test), all of which were significantly lower than those in the choice task (Fig. 6A, *P* <0.001, *Z*-test). Moreover, we found that in the choice task, the percentage of neurons encoding reward amount with a positive slope (30/76, 39.5%) was significantly higher than that with a negative slope (1/76, 1.3%) (Fig. 6B, *P* <0.001, *Z*-test). Meanwhile, there was no such trend among neurons encoding reward amount in the non-preferred hemifield (Fig. 6B, positive slope: 9.2% (7/76); negative slope: 13.2% (10/76), *P* = 0.30, *Z*-test) or among those encoding luminance level (Fig. 6B, positive slope: 9.2% (7/76); negative slope: 9.2% (7/76), *P* = 1, *Z*-test).

We also quantified the variance in neuronal activity accounted for by reward and luminance using the CPD. We applied the regression model to the spike rates estimated with a sliding 200-ms sliding window stepping through time in 25-ms steps. In the choice task, the average CPD for reward amount in the preferred hemifield rose sharply and was sustained throughout the offer-on period while the CPD for stimulus number in the control task remained low (Fig. 6C). In the meantime, the CPD for reward amount in the non-preferred hemifield (Fig. 6D) as well as for stimulus luminance (Fig. 6E) rose more slowly with a more transient temporal profile.

To gain additional insights into how the signals related to reward amount and luminance evolved during the offer-on period, we applied regression analysis with a sliding window (width = 200 ms, step = 25 ms). Standardized regression coefficients (SRCs) for the reward amount in each time step were averaged separately for the offer in the preferred and non-preferred hemifields of each neuron. The results showed that during the offer-on period, the activity of saccade-modulated neurons tended to increase (or decrease) according to the reward amount of the offer in the neuron’s preferred (or non-preferred) hemifield (Fig. 6F), which is consistent with divisive normalization in the LIP [12, 25]. Meanwhile, regression analysis revealed little divergence of the signals related to the stimulus number in the preferred and non-preferred hemifields in the control task (Fig. 6G).

In the group of neurons not modulated by a saccade, the percentage of neurons encoding reward amount or luminance was significantly reduced (Fig. S2A, B) and the strength of encoding was also greatly attenuated (Fig. S2C–G).

### Relationship Between Reward and Luminance Coding

**Fig. 7:**
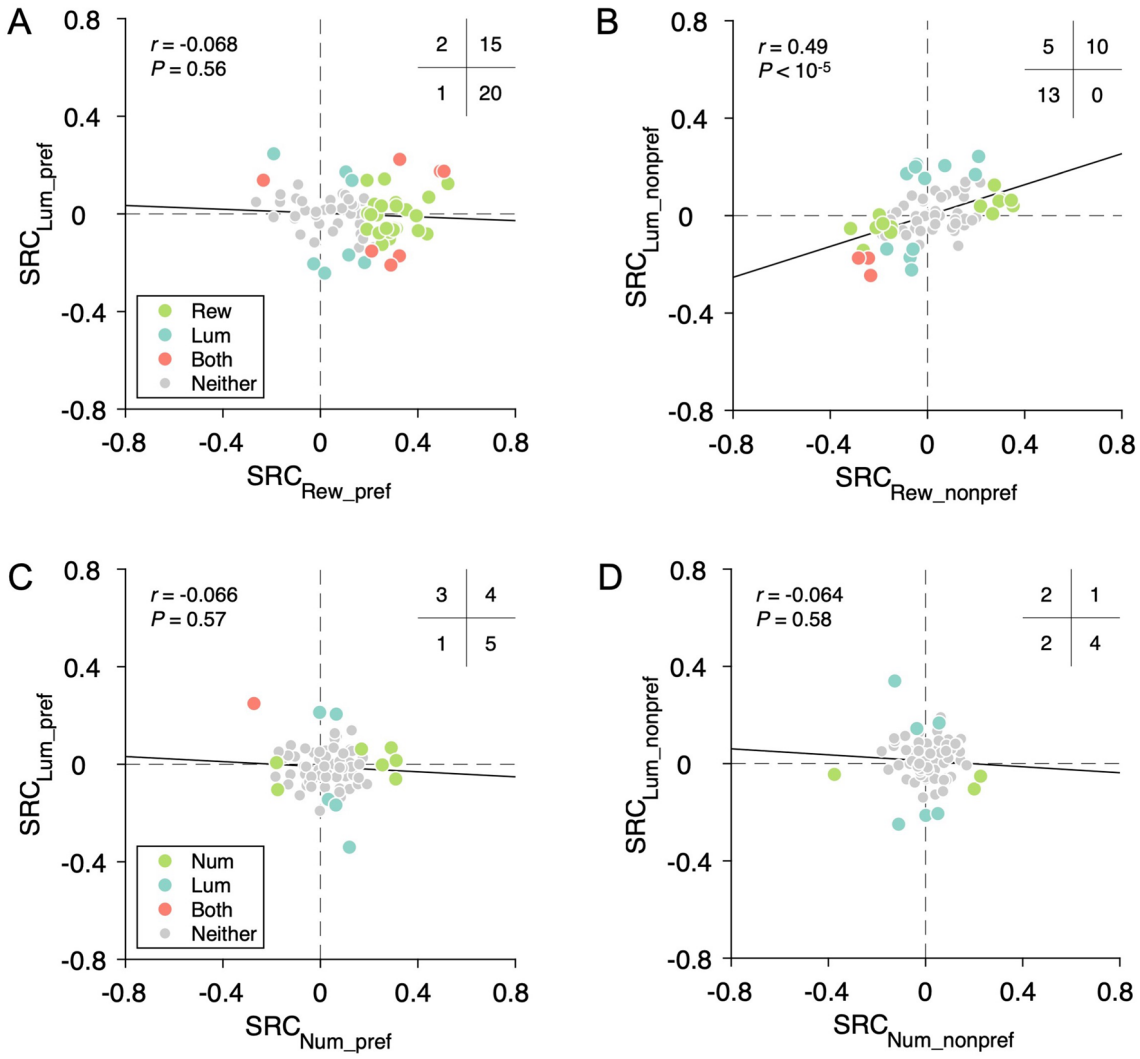
Relationship between reward and luminance encoding. **A** Choice task. No significant association between encoding strength of reward and luminance in the preferred hemifield (*n =* 308, *r* = −0.068, *P* = 0.56, Pearson correlation test). The insert shows the count of neurons encoding reward amount or luminance in each quadrant (*P* = 0.89, Fisher’s exact test). Note that in the regression analysis for this plot, high/low luminance level in the preferred hemifield is coded as 1/0. **B** Choice task. Significant positive correlation between the encoding strength of reward and luminance in the non-preferred hemifield (*n =* 308, *r* = 0.49, *P* <10^−5^, Pearson correlation test). The insert shows the count of neurons encoding reward amount or luminance in each quadrant (*P* <0.05, Fisher’s exact test). Note that in the regression analysis for this plot, high/low luminance level in the non-preferred hemifield is coded as 1/0. **C** Control task. No significant correlation between the encoding strength of stimulus number and luminance in the preferred hemifield in the control task (*n =* 308, *r* = −0.066, *P* = 0.57, Pearson correlation test). The insert shows the count of neurons encoding stimulus number or luminance in each quadrant (*P* = 0.82, Fisher’s exact test). Same convention as in **A**. **D** Control task. No significant correlation between the encoding strength of stimulus number and luminance in the non-preferred hemifield in the control task (*n =* 308, *r* = −0.064, *P* = 0.58, Pearson correlation test). The insert shows the count of neurons encoding stimulus number or luminance in each quadrant (*P* = 0.36, Fisher’s exact test). Same convention as in **C**. SRC, standardized regression coefficient; Rew_pref, reward on the preferred hemifield (choice task); Rew_nonpref, reward on the nonpreferred hemifield (choice task); Lum_pref, luminance on the preferred hemifield; Lum_nonpref, luminance on the nonpreferred hemifield; Num_pref, stimulus number on the preferred hemifield (control task); Num_nonpref, stimulus number on the nonpreferred hemifield (control task).

In light of the priority map hypothesis which suggests the integration of top-down goals with bottom-up saliency, we examined the relationship between reward and luminance encoding. We plotted the SRC for reward amount and luminance in the preferred hemifield against each other. We first tested whether the SRC for reward and luminance tends to have the same sign. We constructed a contingency table counting neurons encoding reward or luminance with different sign combinations, shown as the number of neurons in each of the four quadrants (Fig. 7A, insert). The outcome revealed that there was no significant tendency for neurons to encode reward and luminance with regression slopes of the same sign (*P* = 0.79, Fisher’s exact test). We further found that there was no significant correlation between the SRCs for reward and luminance (Fig. 7A, *r* = −0.068, *P* = 0.56, Pearson’s correlation test). We postulate that these negative results are likely due to the substantial difference in the strength of modulation by reward and luminance in the preferred hemifield (Fig. 6C *vs* 6E).

On the other hand, in the non-preferred hemifield, the strength of modulation for reward and luminance was comparable (Fig. 6D *vs* 6E). We thus investigated the relationship between the encoding of reward and luminance in the non-preferred hemifield. We found a significant tendency for the SRCs for reward or luminance to have the same sign (Fig. 7B, *P* <0.001, Fisher’s exact test). Moreover, there was also a significant positive correlation between the SRC for reward and luminance (Fig. 7B, *r* = 0.49, *P* <10^−5^) and such a positive correlation was evident in both animals (Fig. S3A, monkey F, *r* = 0.56, *P* <10^−4^; Fig. S3B, monkey C, *r* = 0.37, *P* <0.05; Pearson’s correlation test). In the control task, no significant relationship was found between stimulus number and luminance coding in either the preferred or non-preferred hemifield (Fig. 7C, D).

### Distribution of Encoding Neurons Along the Anterior-posterior Axis of the LIP

Previous studies focused on the functional difference between the dorsal and ventral divisions of the LIP in oculomotor control while preliminary evidence implicates functional heterogeneity along the AP axis as well [26]. Taking advantage of the extended AP coverage in our recordings (Fig. 2), we tested whether there is any topographic organization of reward and luminance coding in the LIP.

**Fig. 8:**
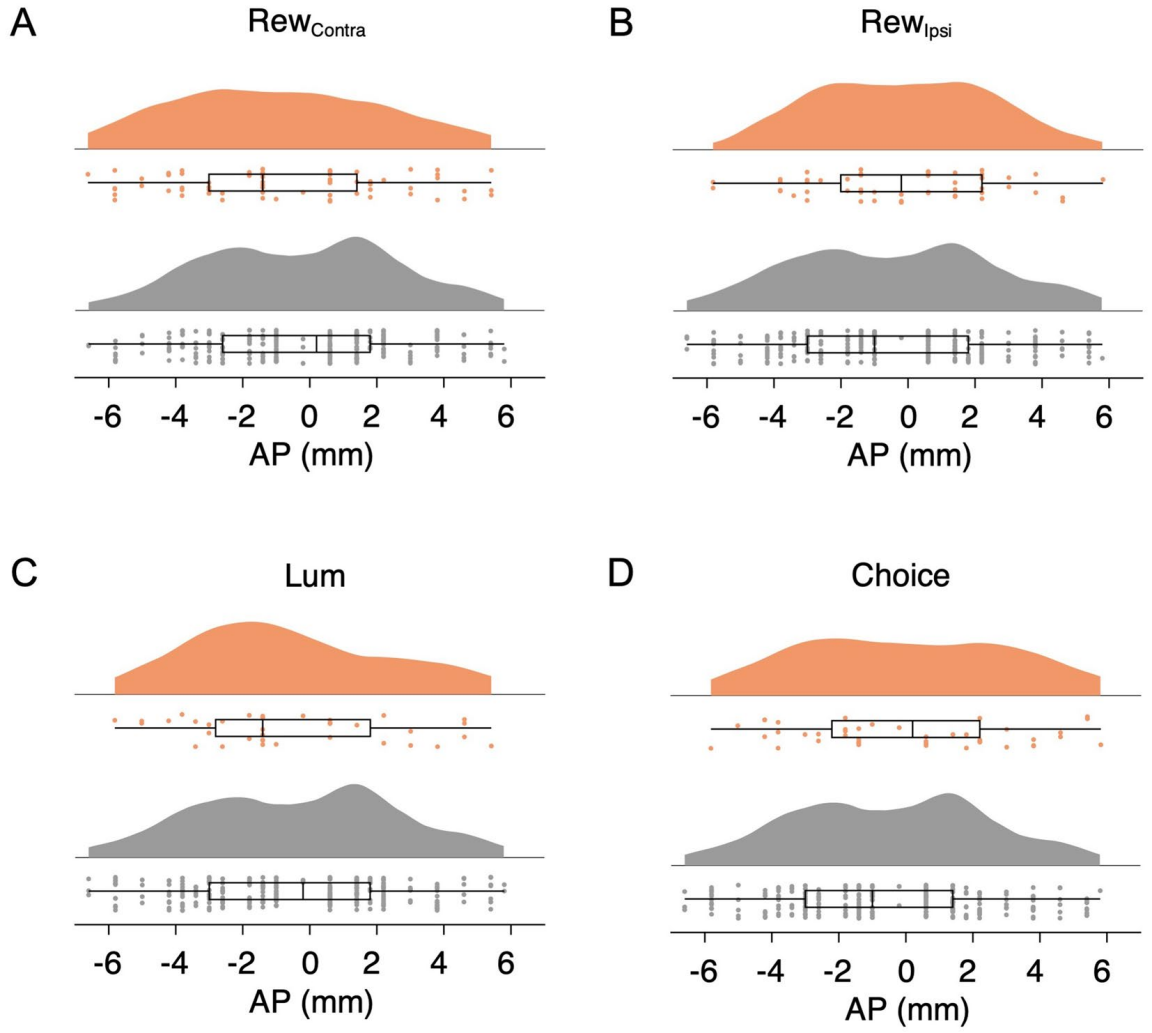
Distribution of AP coordinates of neurons encoding task-related variables in the choice task. **A** No significant difference between the AP coordinate distribution of neurons encoding (top) and not encoding (bottom) Rew_contra_ (top, *n =* 70, bottom, *n =* 238; *P* = 0.20, KS test). **B** As in **A** but for the encoding of Rew_ipsi_ (top, *n =* 53, bottom, *n =* 255; *P* = 0.87, KS test). **C** As in **A** but for the encoding of Lum (top, *n =* 36, bottom, *n =* 272; *P* = 0.53, KS test). **D** As in **A** but for the encoding of Choice (top, *n =* 44, bottom, *n =* 264; *P* = 0.39, KS test).

We compared the distribution of the recording locations along the AP axis for neurons encoding task-related variables with that of the null group in the choice task. The comparison did not yield significant differences regarding reward in the contralateral hemifield (Fig. 8A, *P* = 0.20, KS-test), ipsilateral hemifield (Fig. 8B, *P* = 0.87, KS-test), luminance (Fig. 8C, *P* = 0.53, KS-test), or the animal’s choice (Fig. 8D, *P* = 0.39, KS-test).

## Discussion

In this study, we recorded the activity of LIP neurons while monkeys performed a reward-based choice task with varied reward amounts and luminance levels for the two offers. Neuronal responses were consistent with the choice pattern showing a dominating effect of reward amount and a marginal effect of luminance. First, most of the reward-modulated neurons encoded the reward amount in the neuron’s preferred hemifield with a positive slope, while the composition of positive and negative encoding slopes among luminance-modulated neurons was homogeneous. Meanwhile, in the non-preferred hemifield, the strength of encoding for reward and luminance were positively correlated, suggesting the integration of these two factors in the LIP. We further found that neurons encoding reward and luminance were homogeneously distributed along the AP axis of the LIP. Overall, our study provides further evidence supporting the representation of the priority map in the LIP during reward-based choice.

### Effect of Visual Salience on Reward-driven Choice

A large body of studies has shown that the specific effect of goal-directed attention and bottom-up visual salience on choice behavior depends on the task context. For example, in a consumer choice study, the investigators varied the brightness of the food item display and the display time and the subjects were required to choose as fast as possible with a saccade [27]. They reported that the effect of display brightness on the subjects’ choice decreased substantially with increased display time. Similarly, in a non-human primate study, Markowitz and colleagues [1] designed a two-alternative choice task in which they varied the reward magnitude and luminance of two options trial by trial and the animals were allowed to make a choice immediately after the presentation of these options. They showed that the effect of luminance is dependent on the animal’s reaction time. In particular, when the reaction time is >200 ms, luminance has little effect on the animal’s choice probabilities. The results suggest that when the animals spend sufficient time on deliberation, the reward dominates their choice with little influence of luminance. In another study that also varied visual features indicating reward and perceptual salience, the authors reported that low salience leads to longer reaction times, but not to any change in the choice pattern [28].

The behavioral results in our study are consistent with these findings. In our task, the animal had sufficient time for deliberation since the minimum offer presentation time was 1000 ms. The animal’s choice patterns revealed that offering luminance had a marginal effect. Such a behavioral outcome likely resulted from extensive training such that the effect of higher luminance was attenuated. The neuronal response is consistent with this postulate.

### Effects of Reward and Stimulus Luminance on LIP Activity

We have two main findings regarding the effect of reward and luminance on LIP activity. First, we found that reward and luminance were both encoded in the LIP. This result is consistent with the outcome of a large body of studies reporting the representation of reward or perceptual salience in the LIP [3, 5, 6, 11–13, 15, 29, 30]. Moreover, neuronal activity corresponded with the animal’s choice pattern in that a majority of reward-modulated neurons encoded the reward amount in the neuron’s preferred hemifield with a positive slope while a high luminance level generated a balanced encoding pattern.

Second, we found a significant positive correlation between the encoding of reward amount and luminance. We verified this with two analyses. We first tested among neurons encoding reward or stimulus, whether the encoding slopes for reward and luminance tended to have the same sign. Next, we tested whether there was a significant correlation between the strength of modulation by reward and luminance. Both analyses yielded positive results. However, such a positive correlation only occurred in the neuronal responses to the offer in the non-preferred hemifield. We postulate that this is because, for the preferred hemifield, the strength of modulation for reward amount is substantially stronger than that for luminance (Fig. 6C *vs* 6E) thus dominating the neuronal responses. Meanwhile, more balanced responses in the non-preferred hemifield for reward and luminance (Fig. 6D *vs* 6E) revealed a significant correlation between the encoding strength of these two factors.

To the best of our knowledge, our study is the first to report an interaction of the top-down reward signal with the bottom-up perceptual saliency in the LIP during reward-based choice.

A previous study reported the response of LIP neurons when stimulus luminance alone was manipulated [5]. Tanaka and colleagues recorded from LIP neurons in a visual detection task while manipulating the luminance of visual targets. They found that visual response strength increased and the response onset latency decreased with an increase of stimulus luminance. Furthermore, the luminance-related increase in LIP activity was correlated with a decrease in reaction time. Thus, the LIP response pattern to target luminance is consistent with the task demand of detecting a visual target. However, when bottom-up saliency is incongruent with the task goal, with training, animals can learn to ignore the salient stimulus, and the LIP responses to visually salient distractors are suppressed [30]. Our results are consistent with such findings in that among saccade-modulated neurons, equal proportions of neurons encoded luminance with a positive or negative slope. Thus, the integration of responses across these neurons leads to weak population-level modulation by luminance.

### Anterior-Posterior Distribution of Reward- and Luminance-Coding Neurons

Increasing evidence suggests that the LIP is a functionally heterogeneous area. Non-human primate studies suggest that dorsal-ventral subdivisions of the LIP may be preferentially involved in different aspects of visual-oculomotor processes [19, 20]. Results from non-human primate studies also point to possible subdivisions along the caudal-rostral extent of the LIP [21–23]. It has been reported that electrical stimulation of the caudal and rostral regions of the ventral LIP mostly induces saccades in eye-centered and head-centered coordinates, respectively [26]. In a monkey functional magnetic resonance imaging study, Patel and colleagues reported that based on the measure of blood oxygen level-dependent activity in the ventral LIP, the representation of the fovea (7° eccentricity) is stronger in the rostral region while the representation of the peripheral (15° eccentricity) is stronger in the caudal region [23]. Despite these previous reports, we did not find a significant trend in the distribution of reward- or luminance-coding neurons along the AP axis of the LIP. These results suggest that although there may be a regional difference regarding the reference frame of saccades or the distribution of receptive fields, neurons involved in building the priority map are evenly distributed along the AP axis of the LIP.

## Supplementary Information

Below is the link to the electronic supplementary material.

Supplementary file1 (PDF 279 KB)
