# Supplementary material for: Neuronal Response to Reward and Luminance in Macaque LIP During Saccadic Choice
Source: Neurosci Bull. 2022 Sep 17;39(1):14–28. doi: 10.1007/s12264-022-00948-0 (PMC9849667; doi:10.1007/s12264-022-00948-0)
Supplement: Supplementary file 1 — Supplementary file1 (PDF 279 KB) [file 12264_2022_948_MOESM1_ESM.pdf]

Supplementary Materials

Supplementary Figures

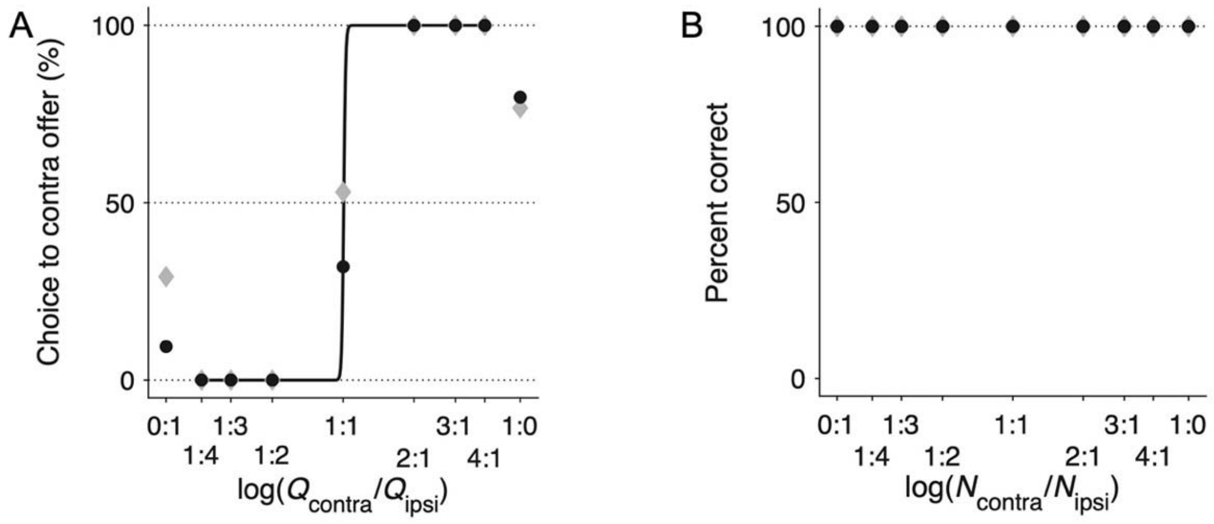

Fig. S1 Choice pattern. **A** Summary choice pattern in the choice task for monkey F under reward matrix #2 (11 sessions, 1443 trials). The percentage of contralateral choices is plotted against $\log(Q_{contra}/Q_{ipsi})$, where Q_{contra} and Q_{ipsi} are quantities of the contralateral and ipsilateral offer, respectively (note: quantity ratios 0:1 and 1:0 are plotted separately thus not on log scale). Trials are separated into two groups depending on the luminance level for the two offers (filled gray diamonds: high luminance on the contralateral offer, filled black circles: high luminance on the ipsilateral offer). The regression lines are from Eq. 1. **B** Summary choice accuracy in the control task for monkey F under reward matrix #2 (11 sessions, 1654 trials). N_{contra} and N_{ipsi} represent the number of stimulus symbols in the contralateral and ipsilateral hemifield (note: number ratios 0:1 and 1:0 are plotted separately thus not on log scale).

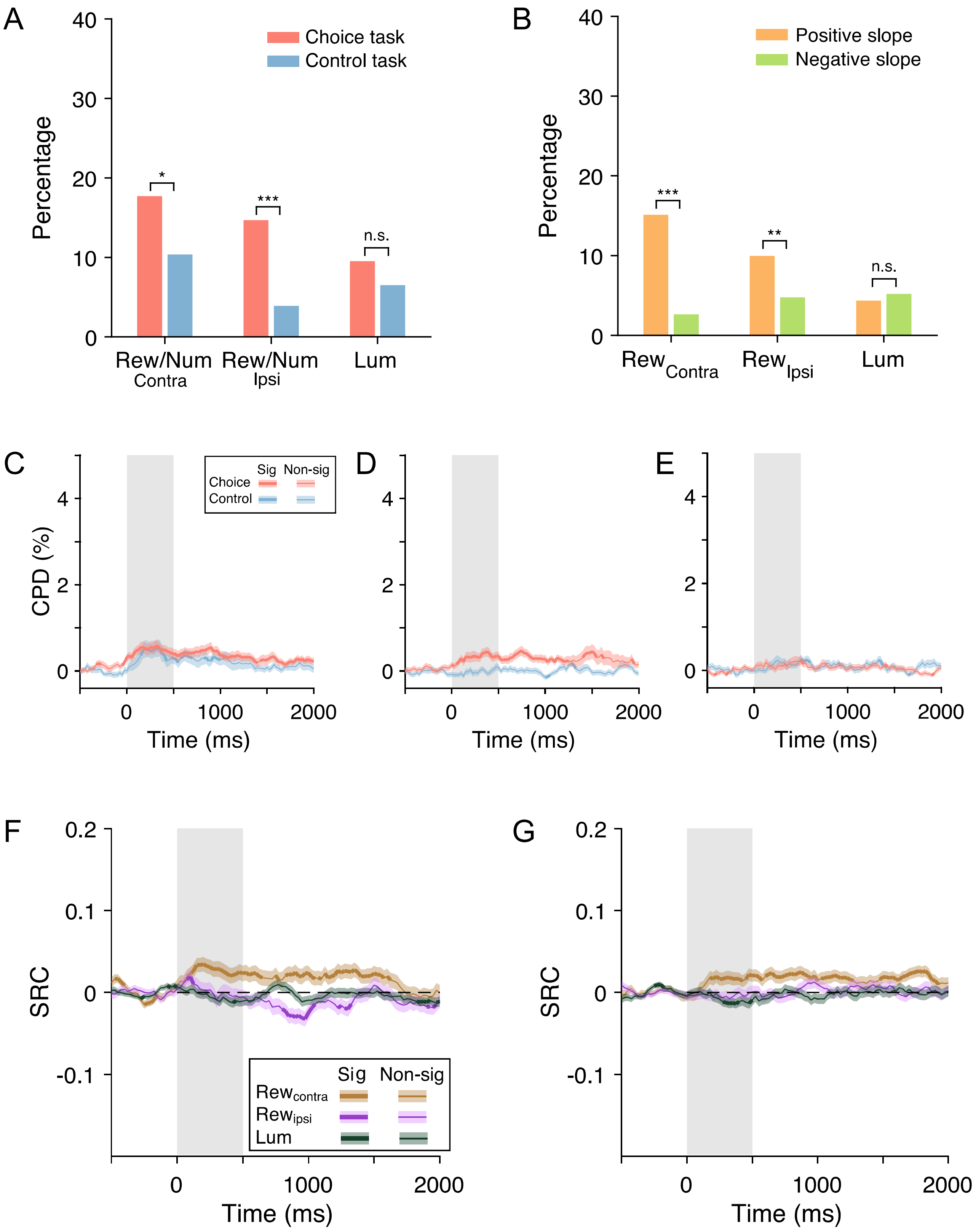

Fig. S2 Encoding of task-related variables in the choice and control task among neurons not modulated by saccades in the control task. **A** Percentages of cells encoding reward (choice task)/stimulus number (control task) and luminance level (**P* <0.05; ****P* <0.001, *Z*-test). Since these neurons were not

modulated by saccades and thus do not have a preferred hemifield, responses to reward amount and luminance are grouped based on the offer's location in the contralateral or ipsilateral hemifield with respect to the recording hemisphere. Rew (Contra, n = 41; Ipsi, n = 34), Num (Contra, n = 24; Ipsi, n = 9); Lum, (Choice task, n = 22; Control task, n = 15). **B** Percentages of neurons encoding reward and luminance with a positive and negative slope in the choice task (**P <0.01; ***P <0.001, Z-test). **C** Time course of neuronal responses related to reward and luminance. Population average of coefficient of partial determination (CPD) for reward amount/stimulus number in the contralateral hemifield in the choice/control task. Each data point was computed based on the firing rate in a sliding time window (width = 200 ms, step = 25 ms). Thick line indicates that the mean CPD is significantly above baseline (P <0.05, t-test). The gray rectangle covers the post-offer window. Shaded regions indicate the mean ± SEM. **D** As in **C** but for reward amount/stimulus number in the ipsilateral hemifield. **E** As in **C** but for luminance. **F** Time course of the standardized regression coefficients (SRCs) for reward and luminance in the choice task (n = 308). Each data point was computed based on the firing rate in a sliding time window (width = 200 ms, step = 25 ms). Thick line indicates that the mean SRC deviates significantly from 0 (P <0.05, t-test). Shaded regions indicate the mean ± SEM. The gray rectangle covers the post-offer window. **G** As in **F** but for stimulus number and luminance in the control task (n = 308).

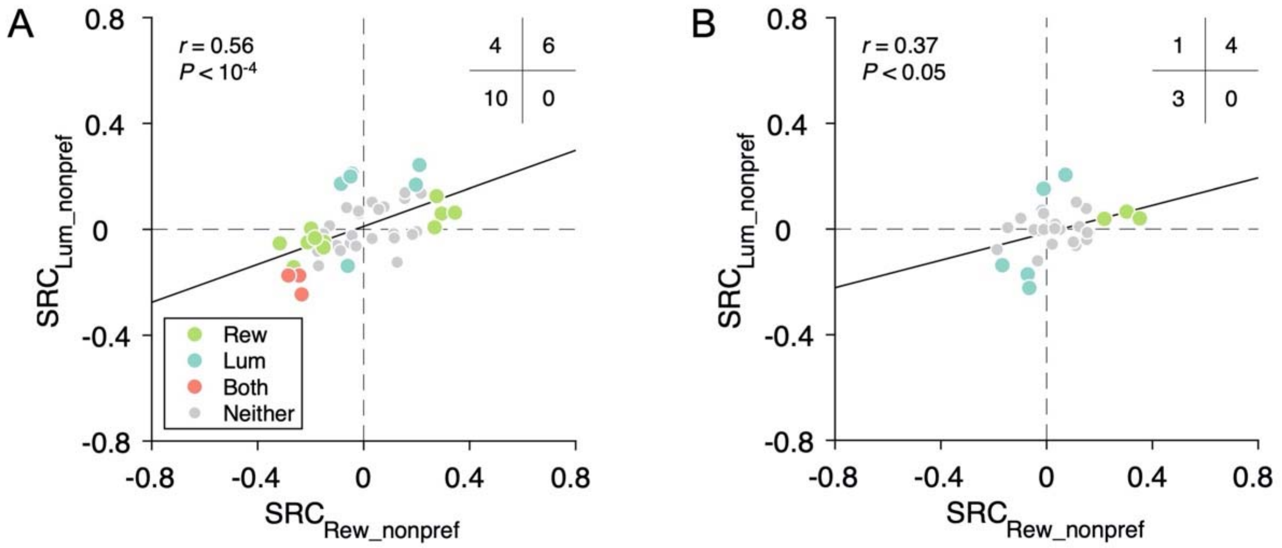

Fig. S3 Relationship between reward and luminance encoding in the choice task for monkey F (**A**) and monkey C (**B**). In both monkeys, there is a significant correlation between the encoding strength of reward amount and luminance in the non-preferred hemifield (monkey F, $n = 180$, $r = 0.56$, $P < 10^{-4}$; monkey C, $n = 128$, $r = 0.37$, $P < 0.05$; Pearson's correlation test). Note that in the regression analysis for this plot, high/low luminance level in the non-preferred hemifield is coded as 1/0.
